# Characterization of peripheral immune cells in kidney transplantation recipients under different immunosuppressive treatments

**DOI:** 10.3389/fimmu.2025.1605664

**Published:** 2025-06-11

**Authors:** Yunze Tai, Nanjing Li, Jiwen Fan, Haohan Zhang, Honghui Long, Lin Yan, Weihua Feng, Junlong Zhang, Bei Cai, Yu Fan, Yao Luo, Yi Li

**Affiliations:** ^1^ Department of Laboratory Medicine, Sichuan Medical Laboratory Clinical Medicine Research Center, West China Hospital, Sichuan University, Chengdu, Sichuan, China; ^2^ Division of Radiotherapy, Cancer Center, West China Hospital, Sichuan University, Chengdu, Sichuan, China; ^3^ Department of Urology, West China Hospital, Sichuan University, Chengdu, Sichuan, China; ^4^ Department of Transfusion Medicine, West China Hospital, Sichuan University, Chengdu, Sichuan, China

**Keywords:** kidney transplantation, immune cell biomarker, myeloid-derived suppressor cells, mTOR inhibitors, flow cytometry

## Abstract

**Background:**

A comprehensive peripheral immune cell characterization including novel immunosuppressive subsets myeloid-derived suppressive cells (MDSCs) in kidney transplant recipients (KTRs) under different immunosuppressive treatments can help: 1) Immunosuppression situation and allograft acceptance assessment; 2) Infection and rejection emergence indication; 3) Beneficial immunosuppressive regimens’ selection.

**Methods:**

26 KTRs with an average transplant duration of 360 days and 13 healthy controls were enrolled in this study. 11KTRs were included in the SRL-based therapy group and the other 15 in the TAC-based therapy group. Flow cytometry was used to detect the percentages and absolute numbers of MDSCs, T cell populations, HLA-DR^+^ monocytes, neutrophil CD64 index, and cytokines in peripheral blood.

**Results:**

In KTRs, the expression of G-MDSCs and M-MDSCs was significantly higher than the HCs, while the expression of HLA-DR^+^ monocytes, CD38^+^/CD28^+^ activated T cells, CD4^+^ naïve T cells, CD4^+^ effector memory T cells, and central memory T cells were significantly lower. The use of mTOR inhibitors in KTRs induced changes in the distribution of activated and naïve-memory T cell subsets and decreased proinflammatory cytokines.

**Discussion:**

In KTRs, G-MDSCs and M-MDSCs accumulated while functionally activated, naïve-memory T cell populations and HLA-DR^+^ monocytes markedly decreased one year after transplantation. Additionally, the number of MDSCs and T cell subsets following transplantation is likely regulated by mTOR inhibitors.

## Graphical Abstract

Summarization of the effects of mTOR inhibitors on KTRs’ peripheral immune cell phenotypes. The figure was created in BioRender.

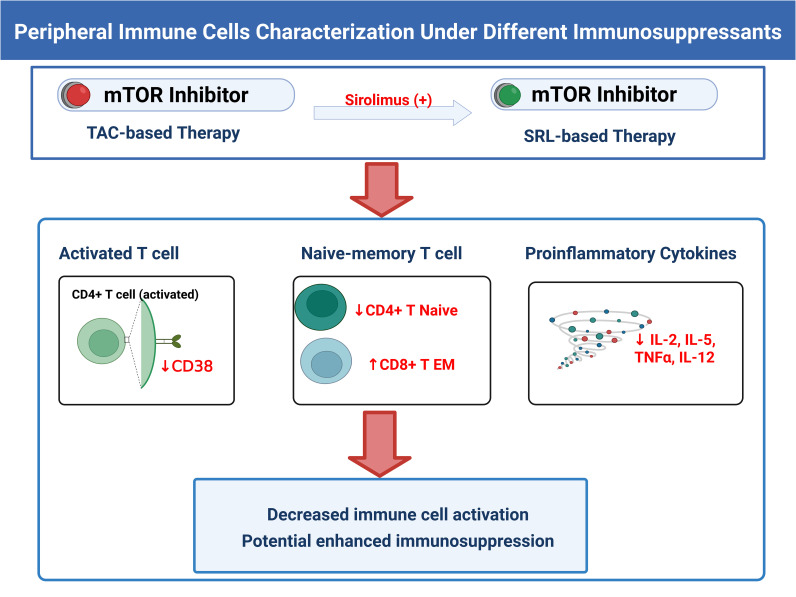

## 1 Introduction

Kidney transplantation has become the only effective treatment for patients with end-stage renal diseases (1, 2). The short-term survival rates of kidney transplantation recipients (KTRs) can be as high as 90%, but long-term survival rates still require improvement due to late graft failure mediated by chronic transplantation rejection or calcineurin inhibitors (CNIs) nephrotoxicity (3, 4). Using the mammalian target of rapamycin (mTOR) inhibitors with low tacrolimus (TAC) concentrations may help lessen the effects of chronic renal damage (4). Rapamycin (also known as sirolimus, SRL) can also suppress the differentiation and proliferation of effector CD8^+^ T cells and the expression of IFN-γ (5, 6), trigger regulatory T cells (Tregs) (7), and negatively affect the proliferation, migration, and antigen-presentation of dendritic cells (DCs) (8). Several studies have examined the efficacy and preservation of graft function between the TAC-based immunosuppressive regimen and SRL-based regimen with reduced CNI exposure, both with maintenance corticosteroids and mycophenolic acid (MPA) (9). However, most of these studies focused on recipients’ kidney function and survival outcomes, with the absence of a comprehensive immune function monitor.

The body’s immune system consists of two parts: innate immunity and adaptive immunity. Both neutrophil CD64 (nCD64) and monocytic HLA-DR (mHLA-DR) can well reflect the body’s innate immune response (10, 11). It is worth noting that MDSCs (myeloid-derived suppressive cells), known as a group of myeloid-derived and heterogeneous cells with immunosuppressive function, have aroused growing research enthusiasm for inducing immune tolerance in transplantation (12–14). Human MDSCs consist of two major groups classified as granulocytic MDSC (G-MDSC: CD11b^+^CD33^+^ CD45^+^CD14^-^CD15^+^HLA-DR^-^) and monocytic MDSC (M-MDSC: CD11b^+^CD33^+^CD45^+^CD14^+^CD15^-^HLA-DR^-^CD16^-^) (13, 15). MDSC mainly inhibits the proliferation, activation and effective function of T cells through the expression of arginase I, induced-nitric oxide synthase (iNOS), TGF-β, IL-10 and many other effectors (16–18). The generation of MDSCs might be useful in pathologies involving excessive immune stimulation, such as transplant rejection. To date, MDSCs have been demonstrated to promote transplant immunological tolerance in the skin (19), allogeneic bone marrow (20) and kidney (21). Interestingly, several investigations have revealed that the introduction of rapamycin can trigger MDSC expression alongside enhancing its immunosuppressive capabilities, which can lead to longer allograft survival (22–24). However, Wu et al. found that inhibition of mTOR with rapamycin decreased the M-MDSCs number in allogeneic skin grafts but had no effect on G-MDSCs (25). Thus, further studies are required to clarify the impact of mTOR inhibitor rapamycin as an immunosuppressive medication on MDSCs, especially in KTRs.

In adaptive immunity, the conventional T cell subsets of naive, central memory (CM), effector memory (EM) and effector T cells were defined based on the expression of CD27 and CD45RA (where naive T cells are CD27^+^CD45RA^+^, central memory T cells are CD27^+^CD45RA^−^, effector memory T cells are CD27^−^CD45RA^−^ and effector T cells are CD27^−^CD45RA^+^) (26, 27). Adding activation markers like CD38 and HLA-DR makes it possible to identify the activated T cell subsets in a variety of situations (28). Furthermore, it has been shown that immunosuppressive intensity and early acute allograft rejection (EAR) risk are related to T cell costimulatory molecule CD28 (29), which can lower the threshold of TCR-mediated T cell activation and boost cell activation and cytokine production (30, 31). Except for T cells, monitoring B cell subsets after transplantation is of great importance, as they can differentiate into plasma cells and produce donor specific antibody (DSA), which leads to the occurrence and development of chronic antibody mediated rejection (CAMR) (32).Apart from immune cells, the cytokines that these cells produce upon activation play a crucial role in controlling both innate and adaptive immunological responses, as well as determining the acceptance or rejection of grafts (33), especially in patients with *de novo* allosensitization (34).

**Figure 1:**
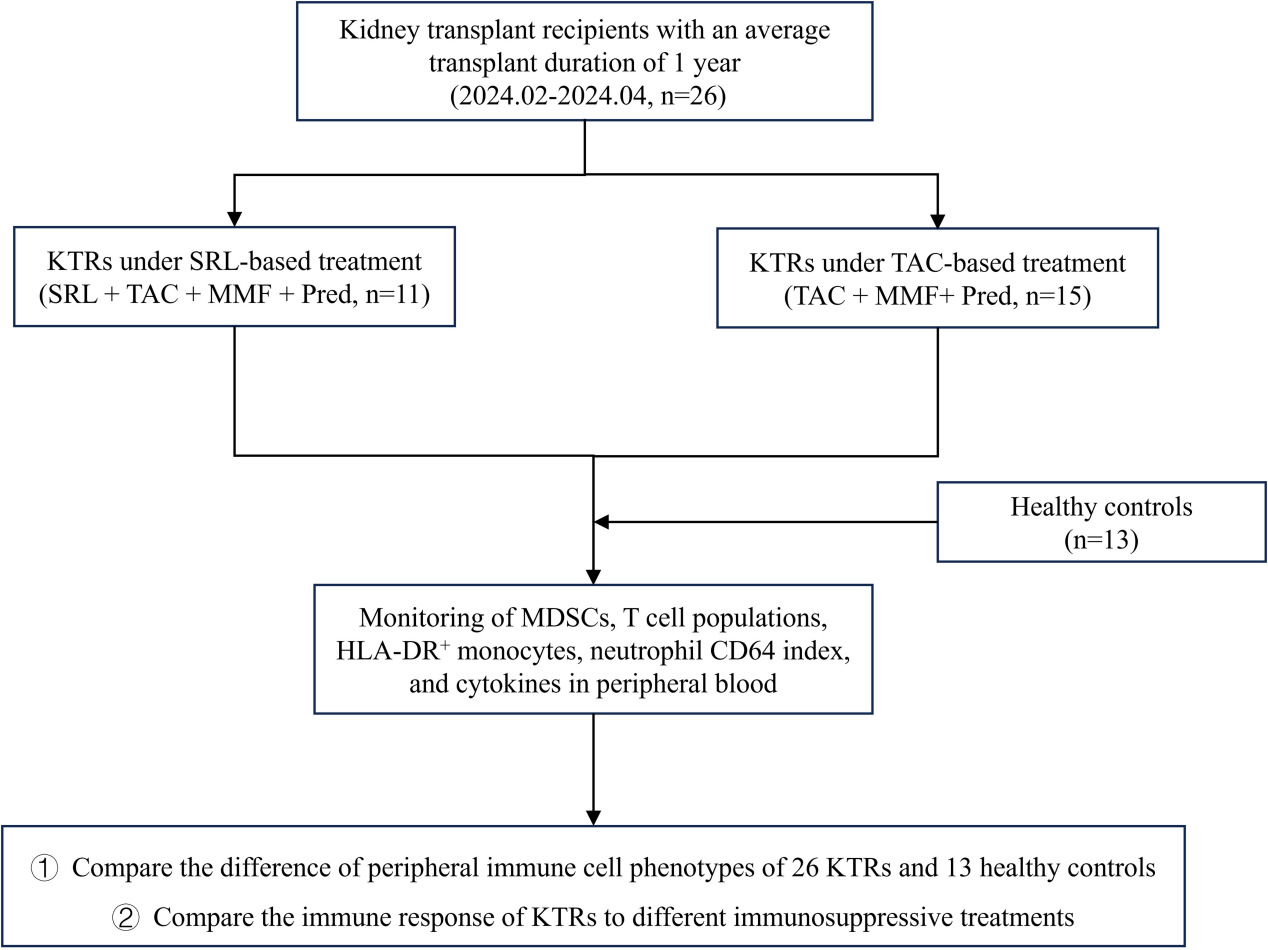
The research flowchart for the sufficient monitoring of immune cell phenotypes in KTRs with different immunosuppressants and HCs. SRL, Sirolimus; TAC, Tacrolimus; MMF, Mycophenolate mofetil; Pred, Prednisolone.

Sufficient real-time monitoring of the body’s immunological status is a prerequisite for the implementation of precise immune intervention in KTRs. However, there is still a lack of systematic monitoring of immune cell subsets as well as a comparative analysis of the effects of the standard triple immunosuppressive regimen and SRL-based quadruple regimen on KTRs’ immune tolerance. To further explore the promising immune biomarkers in KTRs’ prognosis evaluation, we thoroughly examined the immune cell phenotypes of 26 KTRs and 13 healthy controls with flow cytometry analyses. In addition, we also evaluate the efficacy of two different immunosuppressive therapies through these indicators. The research process is shown in **Figure 1**.

## 2 Materials and methods

### 2.1 Patients

A total of 26 adult kidney transplant recipients and 13 healthy controls were prospectively enrolled at West China Hospital between February and April 2024. Kidney transplant recipients included in this study met the following criteria (1): age ≥ 18 years (2), stable allograft function at the time of sampling (3), regular follow-up at West China Hospital with available data on renal function and immunosuppressant trough levels, and (4) comparable baseline characteristics including age, sex, post-transplant time. The exclusion criteria were (1): age< 18 years (2), history of malignancy or autoimmune diseases, or a second kidney transplantation (3), evidence of acute rejection or unstable clinical condition at the time of sample collection. Healthy controls (HCs) were age and sex matched volunteers without a history of chronic kidney disease, immunological disorders, or immunosuppressive therapy.

Peripheral blood samples were drawn on day 360 after the operation. The research was approved by the Ethics Committee of West China Hospital. Written informed permission was acquired by all participants.

### 2.2 KTRs’ immunosuppressive regimen

All patients received induction therapy with rabbit anti-thymocyte globulin (rATG, 1mg/kg per day, 3 days) or basiliximab (20 mg × 2 doses, on days 0 and 4).

In the initial regimen, all patients received calcineurin inhibitor (CNI) tacrolimus, enteric-coated mycophenolate mofetil (MMF) and corticosteroid uniformly since the use of SRL on day 0 can have unaffordable side effects. The tacrolimus dose was adjusted to target C0 concentrations of 6–8 ng/ml during months 0-12, and 5–7 ng/ml thereafter. The initial dose of MMF was 2.0 g/d. Methylprednisolone was given at 7 mg/kg/d on day 0 to day 3, followed by prednisone 60mg/d on the fourth day after surgery and reduced by 10mg daily to 10mg/d. Eleven of the recipients started to receive SRL 1mg/d on day 14 after transplantation, accompanied by an MMF reduction of 0.5g/d, the other 15 recipients remained on the standard triple immunosuppressive regimen.

### 2.3 Flow cytometry analysis

The following panels of fluorochrome-conjugated monoclonal antibodies (mAbs) were utilized in this study: for MDSCs: anti-CD45-BV605 (clone HI30, Cat#2265089), anti-HLA-DR-BV421 (2161612), anti-CD11b-PC7 (1335874), anti-CD33-FITC (1285601), anti-CD16-PE (1307994), anti-CD15-APC (1180500) (all from BD Biosciences). Antibodies were used at a dilution of 1:20 according to the manufacturer’s instructions. G-MDSCs were defined as CD11b^+^CD33^+^CD14^-^CD15^+^HLA-DR^-^CD16^-^ and M-MDSCs were defined as CD11b^+^CD33^+^CD14^+^CD15^-^HLA-DR^-^. The detailed gating strategy is shown in **Supplementary Figure 1** (**Supplementary Figure S1**).

The following mAb panels (all from Beckman Coulter) were used for regulatory T cells (Tregs): anti-CD45-FITC (clone 2D1, Cat#C41137), anti-CD4-PC5.5 (clone 13B8.2, Cat#C41174), anti-CD25-PE (clone B1.49.9, Cat#C41200), anti-CD127-PC7 (clone R34.34, Cat#C41177); for functional activated lymphocytes: anti-CD45-KO (clone J.33, Cat#C41157), anti-CD3-FITC (clone UCHT1, Cat#C41179), anti-CD4-PC5.5 (clone 13B8.2, Cat#C41174), anti-CD8-APC (clone B9.11, Cat#C41165), anti-CD28-PE (clone CD28.2, Cat#C41182), anti-CD38-APC-A750 (clone LS198-4-3, Cat#C41191), anti-HLA-DR-PB (clone Immu-357, Cat#C41192); for naïve-memory lymphocytes subpopulations: anti-CD45-KO (clone J.33, Cat#C41157), anti-CD45RA-FITC (clone 2H4, Cat#C41158), anti-CD4-PC5.5 (clone 13B8.2, Cat#C41174), anti-CD8-APC (clone B9.11, Cat#C41165), anti-CD27-PC7 (clone 1A4CD27, Cat#C41178), anti-CD3-APC-A750 (clone UCHT1, Cat#C41176); for total B cells: anti-CD45-KO (clone SJ25C1, Cat#C41157), anti-CD3-FITC (clone UCHT1, Cat#C41179), anti-CD19-PE (clone 13B8.2, Cat#340364). Antibodies were used at a dilution of 1:20 according to the manufacturer’s instructions.

The following mAb panels (all from BD Biosciences) were used for HLA-DR activation markers on monocytes and CD64 on neutrophils: anti-CD14-PE-Cy7 (clone M5E2, Cat#664139), anti-HLA-DR-APC (clone G46-6, Cat#665330), anti-CD64-PE (clone 10.1, Cat#652830), anti-CD45-PerCP (clone HI30, Cat#664934). Antibodies were used at a dilution of 1:20 according to the manufacturer’s instructions. The flow cytometry tests were performed on the FACSCanto II instrument (BD Bioscience) and DxFLEX (Beckman Coulter), and the results were analyzed with Kaluza V2.1 software.

Results are expressed as percentages of total circulating lymphocytes, or CD3^+^, CD4^+^, CD8^+^ lymphocytes, or CD14^+^ monocytes, and as absolute counts per μL of blood. Besides, the mean fluorescence intensity (MFI) of HLA-DR on gated monocytes was also measured to support quantitative comparisons across groups. The numbers of G-MDSCs and M-MDSCs per μL of blood are derived by calculations based on the leukocyte count. The nCD64 index is calculated as follows


$$\frac{CD64MFI_{PMN}/CD64MFI_{Lym}}{CD64MFI_{MO}/CD64MFI_{PMN}}$$

### 2.4 Cytokine quantification by cytometric bead array

The concentration of IL-1β, IL-2, IL-4, IL-5, IL-6, IL-8, IL-10, IL-12p70, IL-17, IFN-γ, TNF-α, IFN-α was analyzed by cytometric bead array (CBA) multifactor flow detection technology using the manufacturer’s specifications (CellGer Biotechnology, China, Cat#P110100403). In summary, after cytokine standard preparation and cytokine capture bead mixture, 25 μl serum samples from each individual and dilutions for the standard curve were placed in the sample tubes, and the mixed capture beads and phycoerythrin detection reagent were added. After a 2.5-hour incubation and repeated wash steps, the data was collected using FACSCanto II flow cytometer (BD Biosciences, USA) and analyzed using FCAP Array v3.0 (BD Biosciences, USA) to convert fluorescent intensity values to concentrations using a standard curve.

### 2.5 Statistical analysis

Statistical analyses were performed using SPSS (V27.0, SPSS Inc., Chicago, IL, USA) and GraphPad Prism 9 (GraphPad, Inc., La Jolla, CA). The Shapiro-Wilk test was used to perform the normality test on the data. Data conforming to a normal distribution were subjected to t-test analysis, with results being articulated as ± s (mean ± standard deviation). For data that did not follow a normal distribution, the Mann-Whitney U test was employed, and the analysis was conveyed using median values M (Q1, Q3). Outlier detection was performed through the interquartile range (IQR) method. Two-sided P-values ≤ 0.05 were considered statistically significant.

## 3 Results

### 3.1 Patients

#### 3.1.1 Demographic and clinical characteristics of KTRs and healthy controls

We summarized the laboratory data, demographic and clinical characteristics of all participants. The basic characteristics of the KTRs and HCs are summarized in **Table 1**. There were no significant differences in age or sex between the KTRs group and the HCs group. The level of eGFR was significantly lower in the KTRs group than that in the HCs group, and the level of urea and creatinine were significantly higher in the KTRs group than that in the HCs group, demonstrating the serious deficiency of renal function in the KTRs group. Among KTRs, 15 patients were treated with tacrolimus-based triple therapy (TAC + MMF+ Pred), while 11 recipients were given sirolimus-based quadruple therapy (SRL + TAC + MMF + Pred). There were no significant differences between different immunosuppressant groups regarding age, sex, transplant duration, and renal function indices, except the concentration of TAC significantly increased in the TAC-based therapy group. We also found no significant difference in the incidence of transplantation rejection and virus infection between different immunosuppressive medication groups. The baseline characteristics of patients receiving TAC-based triple therapy or SRL-based quadruple therapy are summarized in **Table 2**.

**Table 1:** Demographic and clinical characteristics of 26 KTRs and 13HCs.

Characteristic	KTRs (n=26)	HCs (n=13)	P-value
Age	32.27 ± 8.36	36.23 ± 6.04	0.143
Males	19 (0.73)	8 (0.62)	0.714
Females	7 (0.27)	5 (0.38)	
Time after transplantation (months)	11.94 ± 3.96	/	/
eGFR (mL/min/1.73m^2^)	61.27 ± 23.10	110.48 ± 6.98	0.029*
Urea (mmol/L)	7.70 (6.53, 9.10)	4.50 (3.80, 5.25)	0.000***
Scr (μmol/L)	117.00 (100.75, 160.25)	77.00 (56.00, 79.50)	0.000***

eGFR, estimated glomerular filtration rate; Scr, serum creatinine. *p< 0.05, **p< 0.01 and *** *p*< 0.001.

**Table 2:** Demographic and clinical characteristics of two groups receiving different immunosuppressive medications.

Characteristic	SRL-based Therapy (n=11)	TAC-based Therapy (n=15)	P-value
Age	33.09 ± 9.71	31.67 1.7.52	0.677
Males	8 (0.73)	11 0.64)	0.973
Females	3 (0.27)	4 (0.36)	
Time after transplantation (months)	13.18 ± 5.61	11.03 ± 1.84	0.246
HLA-A mismatches	1 (0.75,1)	1 (1, 1)	0.927
HLA-B mismatches	1 (1,1)	1 (1, 1)	>0.999
HLA-DR mismatches	1 (0.75,2)	1 (1, 1)	0.738
BK-JC status	5 (0.45)	6 (0.4)	>0.999
Infectious complications			
• Fungal pneumonia	2 (0.18)	1 (0.07)	0.556
• Urinary tract infection	1 (0.09)	1 (0.07)	>0.999
• EBV infection	0	2 (0.13)	0.492
eGFR (mL/min/1.73m^2^)	49.28 ± 24.44	70.05 ± 18.18	0.029*
Urea (mmol/L)	12.24 2.10.53	8.19.13.55	0.244
Scr (μmol/L)	227.18 27214.72	117.47 1731.17	0.123
TAC (ng/mL)	5.34 ± 2.12	6.82.81.36	0.039*
SRL (ng/mL)	5.56 ± 1.64	/	/

TA, tacrolimus; SRL, sirolimus. *p< 0.05, **p< 0.01 and ***p< 0.001.

### 3.2 The impact of kidney transplantation and immunosuppressants on the circulating immune cells phenotyping

#### 3.2.1 Innate immune cells

##### 3.2.1.1 Analysis of MDSCs levels in KTRs and HCs

**Figure 2:**
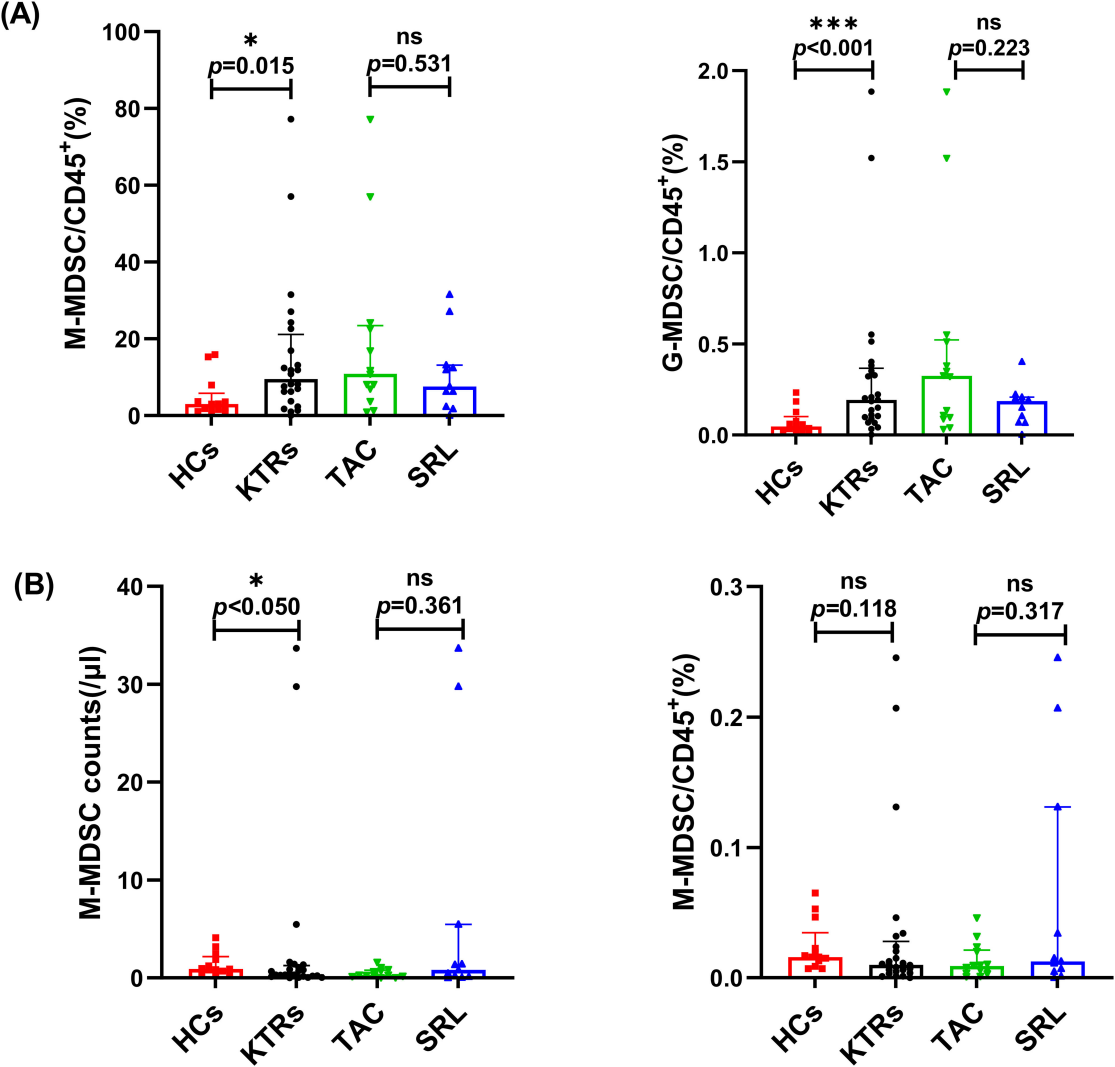
Characterization of circulating MDSC subsets in KTRs applying different immunosuppressants administration and HCs. **(A)** Absolute numbers (left panel) and percentage (right panel) of G-MDSCs in HCs (squares), KTRs (circles), TAC-based therapy group (inverted triangles) and SRL-based therapy group (triangles). **(B)** Absolute numbers (left panel) and percentage (right panel) of M-MDSCs in HCs (squares), KTRs (circles), TAC-based therapy group (inverted triangles) and SRL-based therapy group (triangles). Lines indicate median values including interquartile range (IQR). *p < 0.05, **p < 0.01, ***p < 0.001 calculated with Mann-Whitney U test.

The absolute cell counts and percentage of MDSCs in CD45^+^ white blood cells were compared between the KTRs and HCs groups. As shown in **Figure 2A**, the median absolute number and percentages of G-MDSCs in the KTRs group were much higher than in the HCs group (9.50/μl vs. 3.01/μl, *p* = 0.015; 0.19%, vs. 0.05%, *p<* 0.001, respectively), and the median absolute number of M-MDSCs were much higher than in the HCs group (0.92/μl vs. 0.38/μl, *p<* 0.05 (**Figure 2B**).

Both G-MDSC and M-MDSC levels showed no significant difference between patients who were administered different immunosuppressants (**Figures 2A,B**).

##### 3.2.1.2 Analysis of neutrophil CD64 and monocyte HLA-DR expression levels in KTRs and HCs

**Figure 3:**
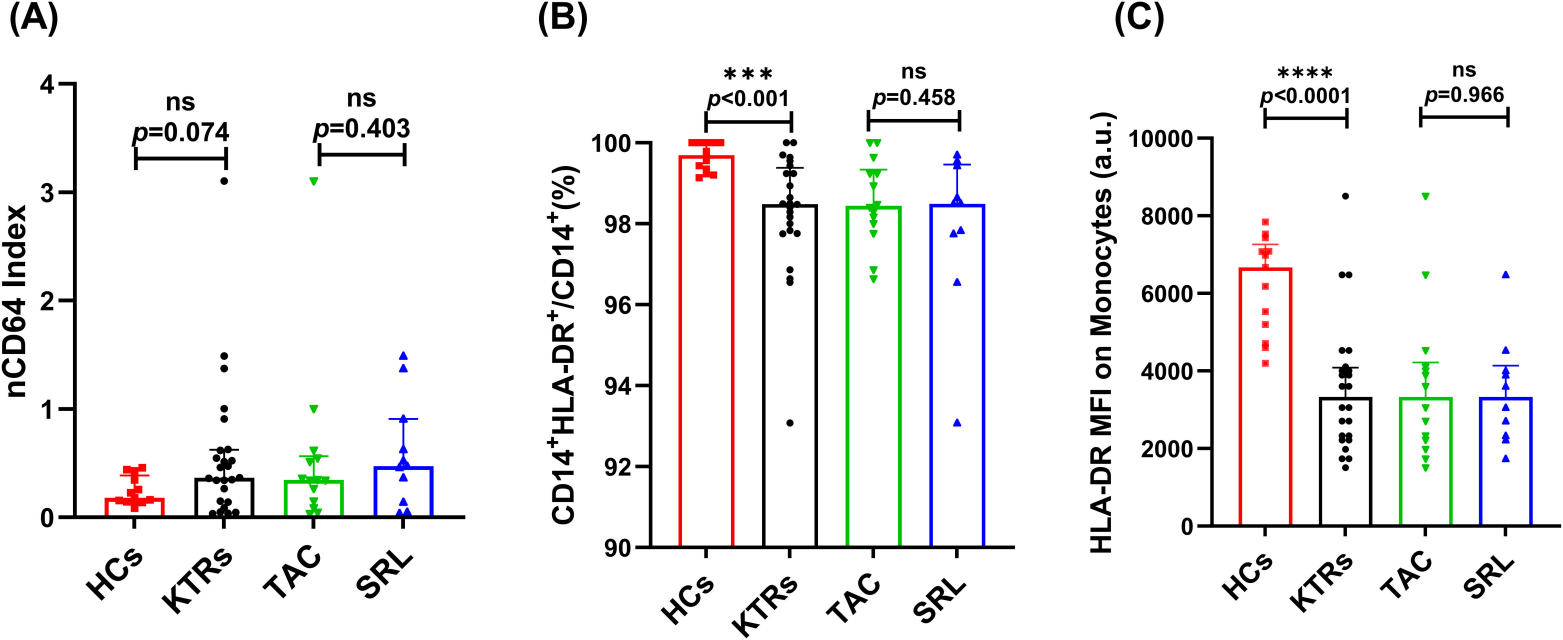
Characterization of nCD64 index and HLA-DR expression on CD14^+^ monocytes in KTRs applying different immunosuppressants administration and HCs. **(A)**nCD64 index in the HCs (squares), KTRs (circles), TAC-based therapy group (inverted triangles) and SRL-based therapy group (triangles). **(B)** Percentage of HLA-DR^+^ monocytes and **(C)** HLA-DR MFI on monocytes in the HCs (squares), KTRs (circles), TAC-based therapy group (inverted triangles) and SRL-based therapy group (triangles). Lines indicate median values including interquartile range (IQR). *p < 0.05, **p < 0.01, ***p< 0.001 calculated with Mann-Whitney U test.

There was no significant difference in the nCD64 index between the KTRs and the HCs group, and a similar situation was observed between the different immunosuppressant treatment groups (**Figure 3A**). However, compared to the HCs group, the median percentage of HLA-DR^+^ monocytes in KTRs was substantially lower (99.69% vs. 98.48%, *p* < 0.001, **Figure 3B**). To complement the percentage-based assessment, MFI of HLA-DR on monocytes was further analyzed. Similarly, a significant downward trend in HLA-DR MFI was observed in KTRs compared to HCs group (3330 vs. 6671, *p* < 0.0001, **Figure 3C**). Additionally, it did not appear that different immunosuppressants had an impact on the monocytes’ HLA-DR expression levels (**Figures 3B,C**).

#### 3.2.2 Adaptive immune cells

##### 3.2.2.1 Regulatory and activated T cells

**Figure 4:**
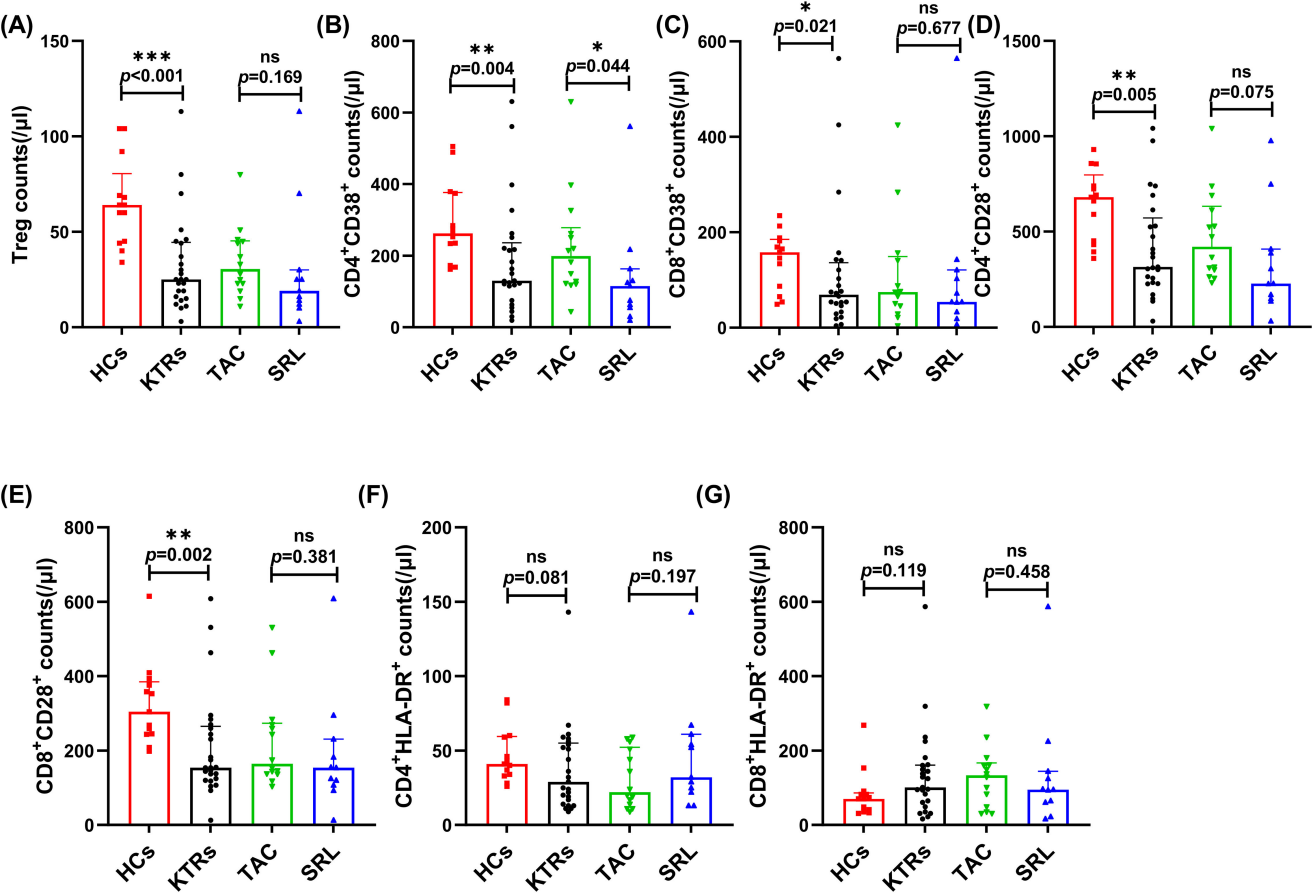
Characterization of circulating regulatory and activated T cell subsets in KTRs applying different immunosuppressants administration and HC. **(A)** Absolute numbers of Tregs in KTRs (circles), HCs (squares), SRL-based therapy group (triangles) and TAC-based therapy group (inverted triangles). **(B)** Absolute numbers of CD38^+^ CD4^+^ T cells, **(C)** CD38^+^ CD8^+^ T cells, **(D)** CD28^+^ CD4^+^ T cells and **(E)** CD28^+^ CD8^+^ T cells, **(F)** HLA-DR^+^ CD4^+^ T cells and **(G)** HLA-DR^+^ CD8^+^ T cells in the HCs (squares), KTRs (circles), TAC-based therapy group (inverted triangles) and SRL-based therapy group (triangles). Lines indicate median values including interquartile range (IQR). *p < 0.05, **p < 0.01, ***p < 0.001 calculated with Mann-Whitney U test.

KTRs had significantly fewer circulating Tregs (25/μl vs. 64/μl, *p* < 0.001, **Figure 4A**) than HCs. However, the median absolute number of Tregs showed no significant difference between various immunosuppressant groups (**Figure 4A**).

The expression level of CD38, CD28 and HLA-DR on the T cell surface indicate their activation status and were measured on T cell subsets in the KTRs and HC groups. The median absolute numbers of CD4^+^/CD8^+^ CD38^+^ T cells and CD4^+^/CD8^+^ CD28^+^ T cells were significantly lower in KTRs than in healthy controls (130/μl vs. 262/μl, *p* = 0.004, **Figure 4B**; 72/μl vs. 158/μl, *p* = 0.021, **Figure 4C**; 315/μl vs. 680/μl, *p* = 0.005, **Figure 4D**; 154/μl vs. 304/μl, *p* = 0.002, **Figure 4E, respectively**), but demonstrated no apparent distinctions between patients receiving various immunosuppressants (**Figures 4B–E**). Noteworthily, patients receiving SRL-based therapy revealed significantly fewer CD38^+^CD4^+^ T cells than those receiving TAC-based therapy (115/μl vs. 199/μl, *p* = 0.044, **Figure 4B**). The expression of HLA-DR on CD4^+^ and CD8^+^ T cells showed no difference (**Figures 4F,G**).

##### 3.2.2.2 Naïve-memory T cells

**Figure 5:**
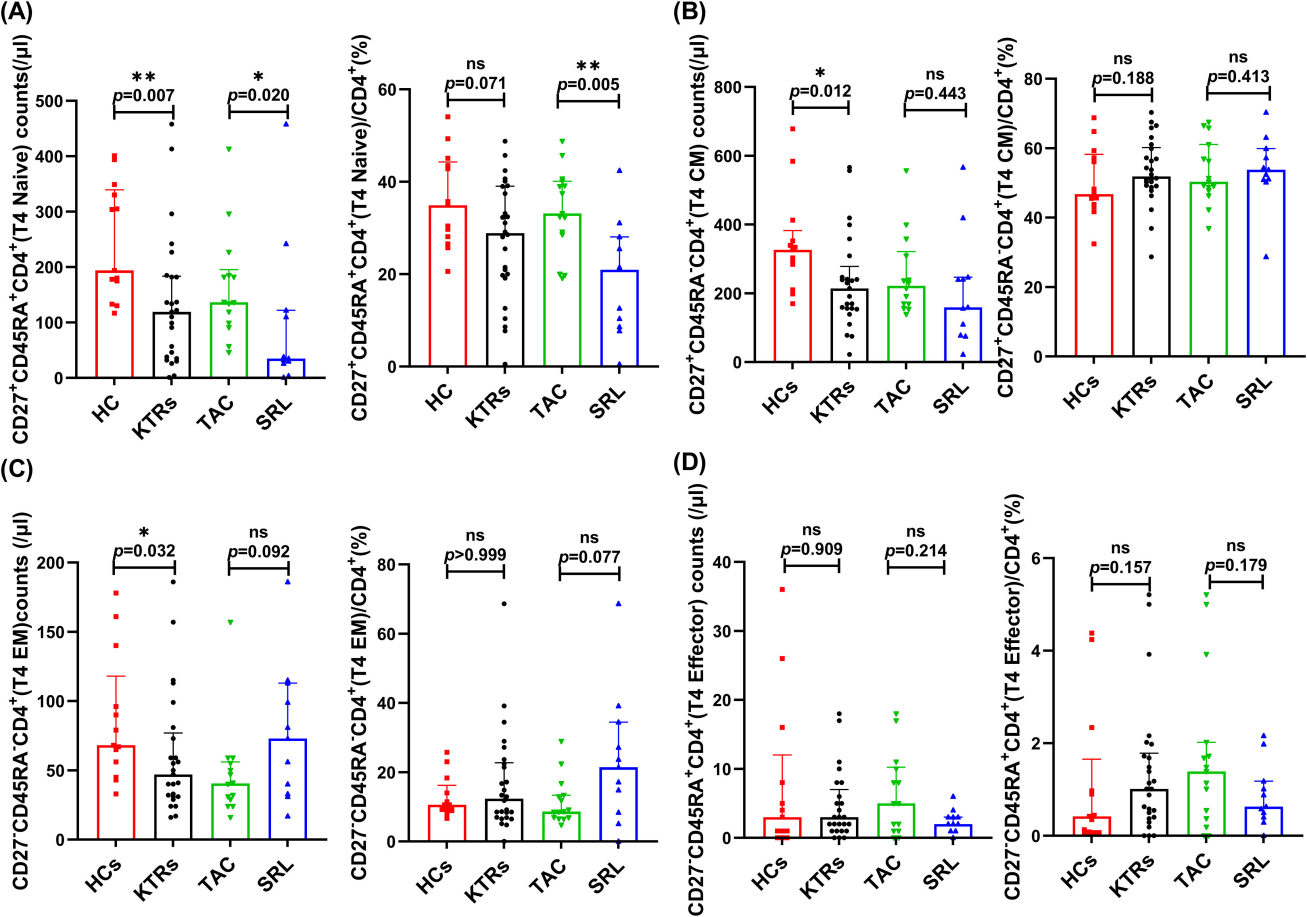
Characterization of circulating CD4^+^ naïve-memory T subsets in KTRs and HCs. **(A)** Absolute numbers (left panel) and percentage (right panel) of CD27^+^CD45RA^+^CD4^+^ naïve T cells, **(B)** CD27^+^CD45RA^-^CD4^+^ central memory T cells, **(C)** CD27^-^CD45RA^-^CD4^+^ effector memory T cells and **(D)** CD27^-^CD45RA^+^CD4^+^ effector T cells in the HCs (squares), KTRs (circles), TAC-based therapy group (inverted triangles) and SRL-based therapy group (triangles). Lines indicate median values including interquartile range (IQR). *p < 0.05, **p < 0.01, ***p < 0.001 calculated with Mann-Whitney U test.

**Figure 6:**
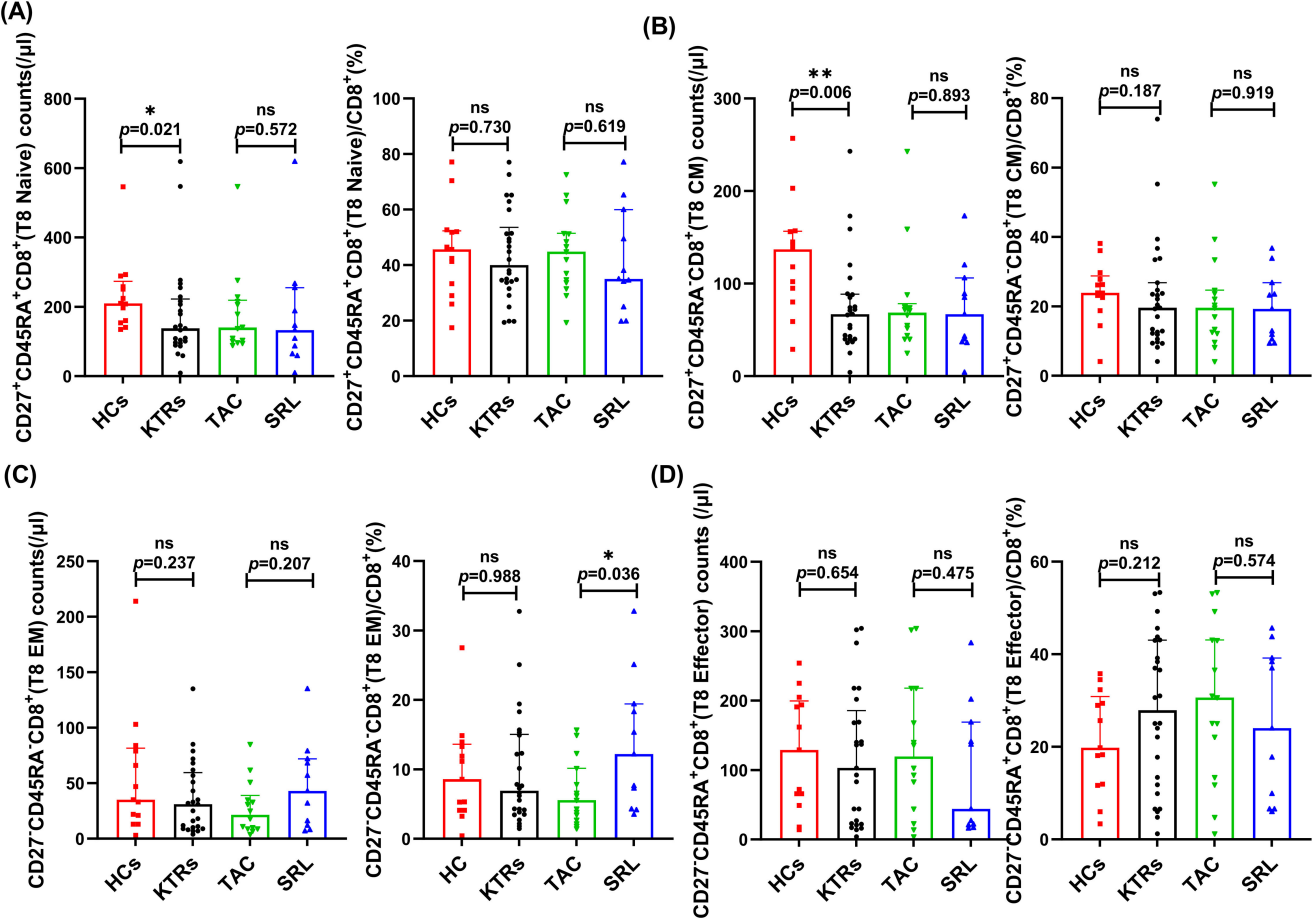
Characterization of circulating CD8^+^ naïve-memory T subsets in KTRs and HCs. **(A)** Absolute numbers (left panel) and percentage (right panel) of CD27^+^CD45RA^+^CD8^+^ naïve T cells, **(B)** CD27^+^CD45RA^-^CD8^+^ central memory T cells, **(C)** CD27^-^CD45RA^-^CD8^+^ effector memory T cells and **(D)** CD27^-^CD45RA^+^CD8^+^ effector T cells in the HCs (squares), KTRs (circles), TAC-based therapy group (inverted triangles) and SRL-based therapy group (triangles). Lines indicate median values including interquartile range (IQR). *p < 0.05, **p < 0.01, ***p < 0.001 calculated with Mann-Whitney U test.

We systematically analyzed the median absolute numbers and percentages of naïve-memory subsets (including naive, central memory, effector memory and effector T cells) of CD4^+^ and CD8^+^ T in KTRs (**Figures 5**, **6**, respectively). Results showed that the median absolute numbers of circulating CD27^+^CD45RA^+^CD4^+^ naïve T cells were significantly lower in KTRs than HCs (119/μl vs. 194/μl; *p* = 0.007, **Figure 5A**). As for memory T cells, KTRs exhibited significantly lower median absolute numbers of CD27^+^CD45RA^-^CD4^+^ central memory T cells, CD27^-^CD45RA^-^CD4^+^ effector memory T cells, and CD27^+^CD45RA^-^CD8^+^ central memory T cells than HCs (214/μl vs. 326/μl, *p* = 0.012, **Figure 5B**; 47/μl vs. 68/μl, *p* = 0.032, **Figure 5C**; and 67/μl vs. 137/μl, *p* = 0.006, **Figure 6B, respectively**). SRL-based therapy group exhibited fewer CD27^+^CD45RA^+^CD4^+^ naïve T cells (35/μl vs. 136/μl, *p* = 0.020; 20.96% vs. 33.13%, *p* = 0.005, **Figure 5A**), but higher CD27^-^CD45RA^-^CD8^+^ effector memory T cells percentage (12.2% vs. 5.56%, *p* = 0.036, **Figure 6C**).

##### 3.2.2.3 Total B cells

**Figure 7:**
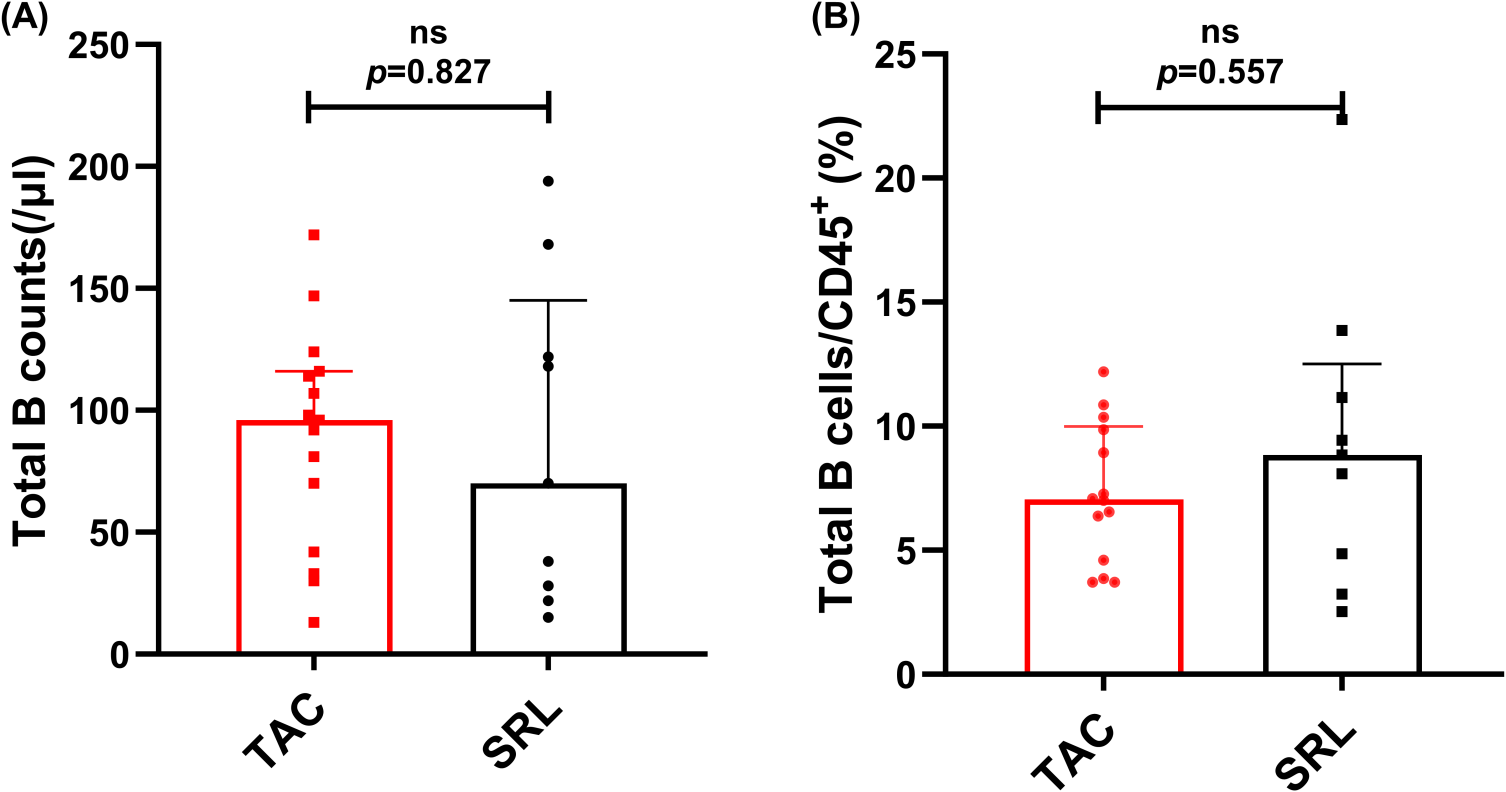
Characterization of circulating total B cells in KTRs under different immunosuppressive treatments. **(A)** Absolute numbers and **(B)** percentage of CD19^+^ B cells in the TAC-based therapy group (squares) and SRL-based therapy group(circles). Lines indicate median values including interquartile range (IQR). *p* was calculated with Mann-Whitney U test.

Median absolute total B cell counts and percentages of CD19^+^ total B cells in lymphocytes were analyzed across KTRs receiving different immunosuppressive therapies (TAC vs. SRL). Results showed that both the median absolute numbers and percentages of total B cells showed no significant difference between various immunosuppressant groups (96/μl vs. 70/μl, *p* = 0.826, **Figure 7A**; 7.05% vs. 8.84%, *p* = 0.557, **Figure 7B**).

### 3.3 Analysis of cytokines levels in KTRs and HCs

**Figure 8:**
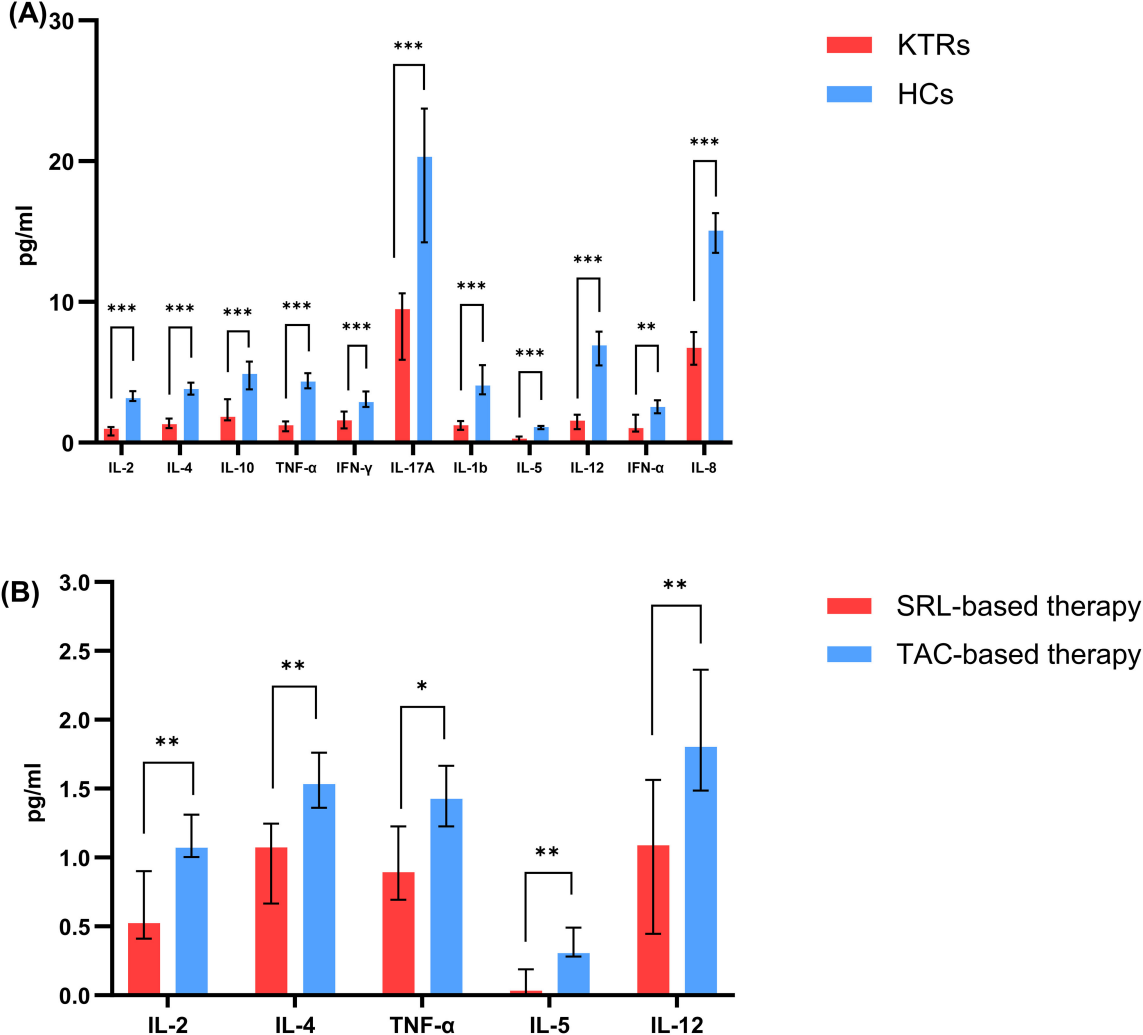
Concentration of circulating adaptive immune cell subsets between patients applying different immunosuppressants administration. **(A)** Concentration of IL-2, IL-4, IL-10, TNF-α, IFN-γ, IL-17A, IL-1b, IL-5, IL-12, IFN-α, IL-8 in KTRs (red histograms) and HCs (blue histograms). **(B)** Concentration of IL-2, IL-4, TNF-α, IL-5, IL-12 in SRL-based therapy group (red histograms) and TAC-based therapy group (blue histograms). Black lines indicate median values including interquartile range (IQR). *p < 0.05, **p < 0.01, ***p < 0.001 calculated with Mann-Whitney U test.

Compared to the healthy controls, the KTRs exhibited considerably lower levels of IL-2, IL-4, IL-10, TNFα, IL-1b, IFN-γ, IFN-α, IL-17A, TGF-b, IL-5, IL-12, and IL-8 (**Figure 8A**, **Supplementary Table 1**). In addition, the SRL-based therapy cohort had lower levels of IL-2, IL-4, TNFα, IL-5 and IL-12 in comparison to the TAC-based therapy cohort (**Figure 8A**, **Supplementary Table 2**).

### 3.4 Summarization of peripheral immune cell subset changes across study groups

**Figure 9:**
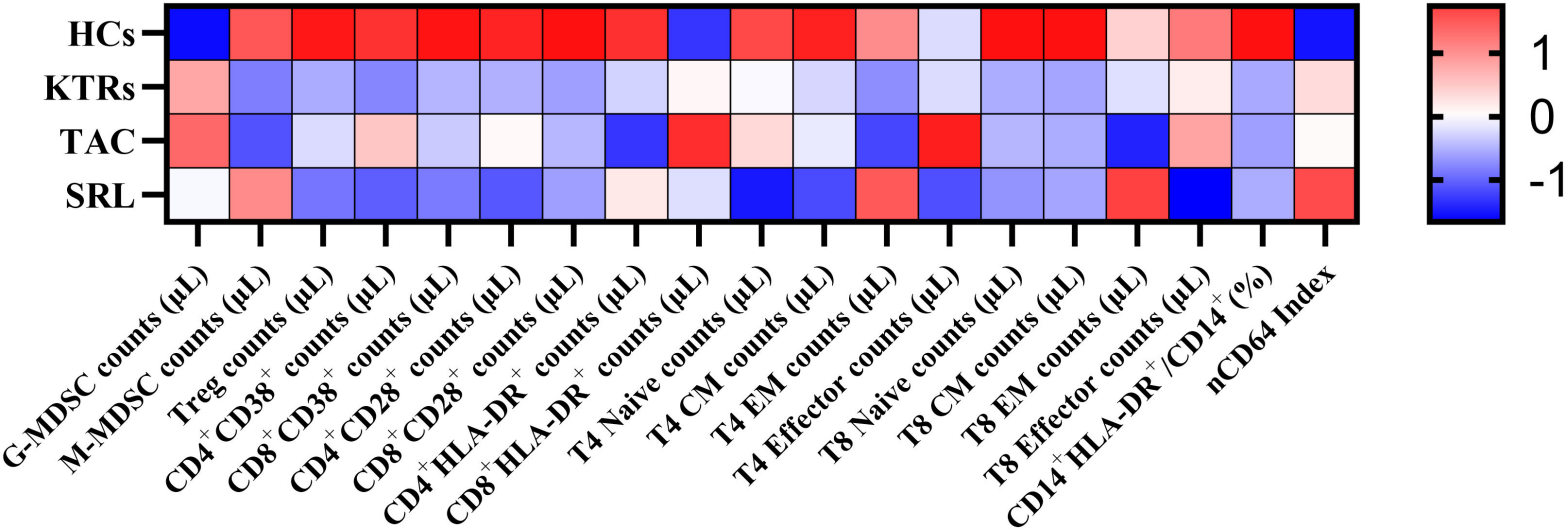
Heatmap of peripheral immune cell subset changes across study groups. The heatmap shows Z-score standardized values of selected immune cell subsets in HCs, KTRs, and immunosuppressive subgroups (TAC, SRL). Immune cell types are grouped into myeloid and T cell lineages. Red indicates relative upregulation; blue indicates relative downregulation.

To provide a comprehensive overview of immune alterations, we summarized all analyzed immune cell populations and their distribution across HCs, KTRs, and the TAC and SRL treatment subgroups (**Figure 9**).

## 4 Discussion

Previous studies have shown that assessing the state of immunosuppression demonstrates benefits in indicating the emergence of microbial infection, inflammation, and transplant rejection resulting from improper clinical or medication treatment approaches in KTRs (35–37). The essential problem is: How can we precisely evaluate the immune status of the KTRs to prevent both allosensitization and risk of infectious complications (38)? Traditional methods for evaluating immunosuppression involve therapeutic medication monitoring and blood routine examination, which are too rough to adequately reflect the immune system’s complexity (37, 39). Immune cell biomarkers including MDSCs, regulatory T cell or follicular T cell subtypes or cytokines such as CXCL13 are gradually being introduced for precise immune monitoring in KTRs (35, 36, 38, 40).

From an immunological point of view, we found that KTRs with an average transplant time of one year manifest more G-MDSCs and M-MDSCs than healthy control. Several studies have pointed out the function and the dynamic changes in the frequencies of MDSCs after human organ transplantation (21, 41, 42). For example, Luan et al. found that MDSC frequencies increased from 3 to 12 months post-transplantation, which is consistent with our findings (43). Hock et al. also identified higher levels of MDSCs frequencies in KTRs with long-term transplants (median = 10 years) than in healthy donors (36, 44). Notably, KTRs complicated with cutaneous squamous cell carcinoma (SCC) showed an increase in G-MDSCs, suggesting that G-MDSCs represent a potential biomarker for immunosurveillance and prognosis-like cancer occurrence in KTRs. However, another study, which included 29 KTRs, showed that M-MDSCs rather than G-MDSCs made up the majority of the MDSCs that accumulated in KTRs’ peripheral blood one year after transplantation (43). In another long-term retrospective study, KTRs with more than 10-year-transplantation and almost-tolerant grafts showed significantly higher M-MDSCs than KTRs with less than 3-year-transplantation, and the M-MDSC levels were positively correlated with the survival rates (45). Therefore, the short-term and long-term changes of different subgroups of MDSCs after renal transplantation may play important roles in mediating transplant tolerance and can be potentially used for recognizing the occurrence of infection and tumor in the prognosis of KTRs.

In addition to MDSC’s negative immunomodulatory effects, monocyte-mediated innate immunity is crucial for antigen presentation, T helper cell activation, and ultimately adaptive immunity that results in graft rejection (46). The antigen presentation function of monocytes is closely related to the expression level of HLA-DR (11), and the decreased expression of HLA-DR in monocytes (mHLA-DR) is related to immunosuppression (47). Monocyte infiltration and HLA-DR expression were observed to increase in allograft biopsies of patients experiencing acute rejection (48, 49). Additionally, monocyte-derived chemotactic transcripts, including tumor necrosis factor (TNF-A) and macrophage inflammatory protein (MIP)-1a amplification (50), were also found to be elevated and were linked to graft loss. Our results revealed that the expression of monocytes’ HLA-DR in KTRs was substantially lower than in HCs, suggesting a greater immunological tolerance for patients in KTRs. Notably, increased expression of complement regulatory proteins (e.g. CD35, CD55, CD46, and CD69) on neutrophils and monocytes has been associated with bacterial and viral infections (51). Although these indicators may help determine infection risk in KTRs receiving different immunosuppressants, they were not assessed in this study, which is a limitation.

Activation markers including HLA-DR and CD38 for immune cells provide information about KTRs’ reaction to infection or cancer, and the combination of CD38 and HLA-DR proved to help detect activated T cells. CD38 metabolizes NAD^+^ to adenosine diphosphate ribose or cyclic ADP ribose and nicotinamide, and may act as an adhesion molecule (interacting with CD31) and a cell-activating receptor that triggers cell proliferation and inflammatory cytokine production after ligation (52). As a secondary signal, T cell co-stimulatory molecule CD28 can lower the threshold of TCR-mediated T cell activation, which plays a key role in the complete activation of T cells (30, 31). Loss of CD28 on peripheral T cells is one of the characteristics of T cell senescence (53), which decreases the risk for early acute rejection after kidney transplantation (29). Our results revealed that KTRs manifest significantly lower CD4^+^/CD8^+^ CD38^+^ T cells and CD4^+/^CD8^+^ CD28^+^ T cells activated T cells than healthy controls, indicating KTRs in our cohort manifested weaker immune cell activity, higher degree of immunosuppression and a lower likelihood of allograft rejection. The pathology findings also demonstrated that the patients had no signs of rejection. Treg cells are responsible for immunosuppressive regulation. KTRs had significantly fewer circulating Tregs than HCs, due to tacrolimus suppressed IL-2 production and downstream of Foxp3 expression, resulting in impaired Treg cell generation (54).

KTRs tended to have a declined abundance of CD4^+^ naïve T cells, CD4^+^ T_EM_ cells, CD4^+^ T_CM_ cells and CD8^+^ T_CM_ cells compared to HCs. Naive T cells are responsible for reacting to unknown antigen stimulation and differentiating into effector cells and memory cells. Memory cells located either in secondary lymphoid organs (T_CM_) or peripheral tissues (T_EM_) are capable of rapid immune responses to known antigens by proliferating and differentiating into effector T cells as well as synthesizing a variety of inflammatory cytokines and cytolytic effectors (55). These results may indicate that KTRs manifest defective generation of immunity to newly encountered antigens, resulting in an increased risk of infection and cancer.

Importantly, T follicular helper (T_FH_) cells are a subset of CD4^+^ T cells essential for B cell activation and differentiation, while follicular regulatory T (T_FR_) cells suppress excessive T_FH_ activity to maintain humoral immune balance. T_FH_ or T_FR_ cells have been increasingly recognized as central regulators of humoral immunity. Previous studies have demonstrated that T_FH_ cells stimulate B cell differentiation and donor-specific antibody (DSA) production through IL-21 secretion, and an imbalance between T_FH_ and T_FR_ cells has been associated with the development of *de novo* DSA (dnDSA) and antibody-mediated rejection (AMR) (34, 56). *In vitro* alloreactivity assay also found the donor-specific HLA reactive TH cell pool increased post-transplant and remained elevated up to one year, while the and allospecific IL-21/IL-17A/IFN-γsecretion showed no significant changes (34). Although we did not include a dedicated analysis of T_FH_ or T_FR_ cells in this study, our previous work demonstrated that increased proportions of PD-1^+^ICOS^+^ and CD226^+^/TIGIT^+^ T_FH_ cells are associated with humoral immune dysregulation in patients with chronic antibody-mediated rejection (CAMR) (57). These findings support that T_FH_ cells need further investigation as a potential diagnostic or therapeutic target. The fine immune phenotyping and functional assays to characterize the T_FH_–T_FR_ balance and its impact on B cell responses and KTRs’ clinical outcomes should be carried out in our future studies.

Furthermore, it’s critical to understand how different immunosuppressive medications especially mTOR inhibitors affect immune cells in KTRs for creating tolerance promotion therapies. In this study, we examined the immune cell phenotypes of 11 KTRs under mTOR inhibitor (SRL) and 15 KTRs under calcineurin (TAC) at 12 months of immunosuppressive therapies after transplantation. Although our findings show no significant difference in both G-MDSC and M-MDSC levels between patients applying different immunosuppressive administrations, G-MDSC levels from KTRs under SRL treatment demonstrated a decreasing tendency, while M-MDSC levels demonstrated a growing tendency compared to KTRs under TAC treatment. Some previous studies have confirmed that SRL promotes the expansion, recruitment and immunosuppression of MDSCs by inhibiting the mTOR signaling pathway (22, 58). For example, SRL prolonged the survival duration of cardiac allografts by enhancing MDSC migration and suppressive action (23). Zhang et al. found that SRL facilitates the recruitment of M-MDSC to protect against murine immunological hepatic injury (58), which is in line with our findings. Other research, however, confirmed that SRL significantly decreases the cell number and the M-MDSCs’ immunosuppressive ability of T cells in allografts-transplanted mice because mTOR is essential for M-MDSC differentiation and immunosuppressive function (25). A previous study revealed that MDSCs under TAC immunosuppressant maintenance for one year, but not SRL, were able to reduce effectively CD4^+^ T cell proliferation *in vitro* (42). In summary, how different immunosuppressive medications regulate the number and functionality of MDSC subgroups in KTRs needs further exploration.

Besides focusing on the important immunosuppressive MDSCs, we also found that KTRs under SRL treatment had higher CD8^+^ T_EM_ cells, but fewer CD38^+^ activated and CD27^+^CD45RA^+^ naïve CD4^+^ T cells. Compared to naive T cells, alloreactive memory T cells can react to alloantigen more quickly and effectively through altering the speed, location, and effector mechanisms by which alloreactive T cells mediate allograft rejection (59, 60). These findings might suggest that compared to the TAC-based group, CD8^+^ T cells in the SRL-based group had a higher propensity to become activated to alloantigen and trigger allograft rejection, while CD4^+^ T cells in the SRL-based group appeared the opposite tendency. Notably, Bak et al.’s research revealed that SRL inhibited naive T cells while preferentially enhancing the function of effector memory T cells specific to CMV (61). Additionally, Turner et al. discovered that in rhesus monkeys, mTOR inhibitors increased the CD8^+^ central and effector memory T cell responses specific to vaccinia (62). This may explain and support the results of our study. Mechanistic studies explained that SRL promotes the intensity of CD8^+^ central memory T cells and their antigen-specific response in the context of infection through increasing CD62L expression (63, 64). However, other studies proposed that SRL failed to enhance CD8^+^ central memory T cell differentiation when CD8^+^ T cells were stimulated in the context of transplantation (65). Thus, more research is necessary to reveal the molecular signals underlying these distinct CD8^+^T cell responses to mTOR inhibition in transplantation.

Besides, accumulating evidence suggests that the function of T_FH_ cells is significantly influenced by immunosuppressive regimens in kidney transplantation (38). In our previous study, we found that TAC and SRL inhibit T_FH_ cells development and IL-21 expression. Notably, SRL exhibited a stronger suppressive effect on T_FH_ cells compared to TAC, partially attributed to decreased STAT3 signaling (66, 67). Although these functional assays were not carried out in the present study, we recognize the importance of further validating these findings, and we plan to include donor-specific peptide stimulations and follow-up T_FH_/T_FC_ phenotyping, proliferation and cytokine profiling in our future studies across different treatment subgroups in KTRs, including comparisons with healthy controls.

As for CD19^+^ B cells, no significant difference was observed between the TAC and SRL groups. However, detailed phenotyping of B cell subsets—such as naïve B cells (CD27^-^IgD^+^), transitional B cells (CD24^hi^CD38^hi^), memory B cells (CD24^hi^CD38^-^) and plasmablasts (CD27^hi^CD38^hi^) (68), was not assessed in this study, which represents a limitation and needs further investigation. Previous studies have shown that clinically stable KTRs exhibit significant differences in total B cells, transitional B cells, and plasma cells compared to healthy controls (69). TAC and SRL are associated with reduced transitional B cells (70). Further analysis of B cell subsets, particularly transitional B cells, is needed to clarify their clinical relevance.

Additionally, we discovered that a series of proinflammatory cytokines including IL-2, TNF-α, IL-12, and IL-5 in the SRL-based group consistently decreased in contrast to the TAC-based group, which might suggest an increased state of immunosuppression in KTRs under SRL treatment. However, this study still had some limitations. Firstly, the sample size of this study is relatively small and all patients were recruited from a single center. Secondly, the follow-up time is relatively too short for pathological rejection observation in KTRs. Moreover, only the phenotypes of MDSCs and other functional immune cells were examined, but the *in vitro* function assay was not carried out due to clinical limitations, including the insufficient peripheral blood samples from KTRs for sorting a larger quantity of pure MDSCs, the ethical considerations associated with patient sample usage, and the constraints of our laboratory facilities and resources.

## 5 Conclusion

In this study, we found that G-MDSCs and M-MDSCs persistently increased while functionally activated T cell counterparts significantly reduced in KTRs. Also, mTOR inhibitors probably play a role in regulating the numbers of MDSCs and T cell subsets distribution after transplant. Given the therapeutic significance of MDSC-mediated immunological tolerance in the treatment of graft rejection, further studies are required to determine whether MDSC subsets can be included as a promising immunosuppressive biomarker responsible for allograft acceptance in clinical tests as well as manipulated to enhance clinical outcomes. Additionally, how different immunosuppressive medications, especially mTOR inhibitors, affect immunological biomarkers’ proliferation and activity, including MDSCs in KTRs needs further investigation.

## Data Availability

The original contributions presented in the study are included in the article/**Supplementary Material**. Further inquiries can be directed to the corresponding authors.
