# Supplementary material for: Characterization of peripheral immune cells in kidney transplantation recipients under different immunosuppressive treatments
Source: Front Immunol. 2025 Jun 11;16:1605664. doi: 10.3389/fimmu.2025.1605664 (PMC12187787; doi:10.3389/fimmu.2025.1605664)
Supplement: Supplementary file 1 [file DataSheet1.docx]

Supplementary Material

# Supplementary Figures and Tables

## Supplementary Figures


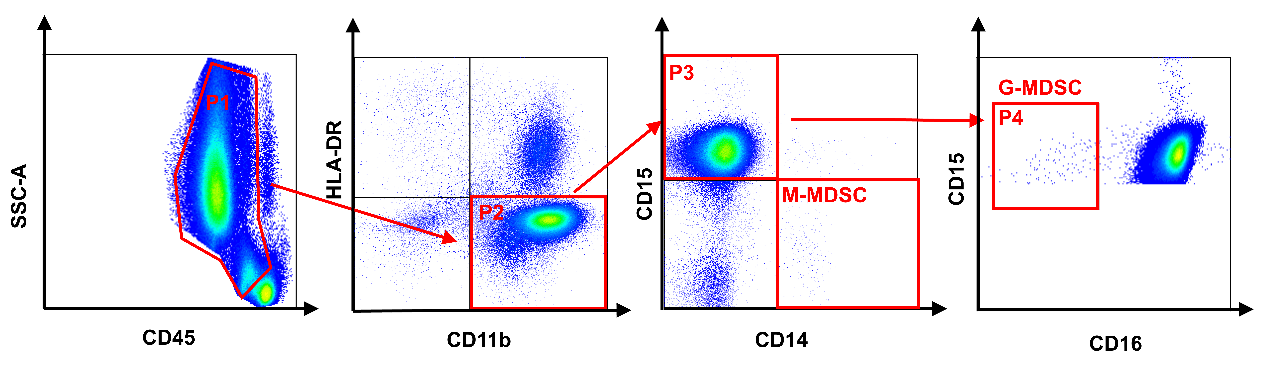


**Supplementary Figure 1.** Staining profiles of MDSCs (CD11b^+^CD45^+^HLA-DR^−^), M-MDSCs (CD11b^+^CD45^+^HLA-DR^−^CD14^+^CD15^−^), and G-MDSCs (CD11b^+^CD45^+^HLA-DR^−^CD14^−^CD15^+^CD16^−^) from representative KTRs and HCs. Measurements were performed with fresh whole blood samples.

## Supplementary Tables

**Supplementary Table 1.** Cytokine quantification of 26 KTRs and 13HCs

|  | KTRs(n=26) | HCs(n=13) | P-value |  |
| --- | --- | --- | --- | --- |
| IL-2 | 0.99(0.52, 1.12) | 3.16(2.97, 3.66) | <0.001 |  |
| IL-4 | 1.30(1.03, 1.72) | 3.82(3.40, 4.25) | <0.001 |  |
| IL-10 | 1.84(1.60, 3.10) | 4.89(3.78, 5.77) | <0.001 |  |
| TNF-α | 1.23(0.81, 1.51) | 4.33(3.85, 4.93) | <0.001 |  |
| IFN-γ | 1.59(1.02, 2.21) | 2.53(2.88, 3.63) | <0.001 |  |
| IL-17 | 9.50(5.90, 10.61) | 20.31(14.25, 23.73) | <0.001 |  |
| IL-1b | 1.24(0.92, 1.54) | 4.06(3.43, 5.51) | <0.001 |  |
| IL-5 | 0.28(0.02, 0.44) | 1.12(0.95, 1.19) | <0.001 |  |
| IL-12 | 0.97(0.97, 2.00) | 6.92(5.49, 7.89) | <0.001 |  |
| IFN-α | 1.03(0.78, 2.00) | 2.54(2.09, 3.01) | <0.001 |  |
| IL-8 | 6.75(5.55, 7.86) | 15.05(13.49, 16.32) | <0.001 |  |

Median and interquartile range from cytometric bead array are displayed. All parameters are expressed in pg/mL.

**Supplementary Table 2.** Cytokine quantification of two groups receiving different immunosuppressive medications

|  | SRL-based Therapy  (n=11) | TAC-based Therapy  (n=15) | P-value |
| --- | --- | --- | --- |
| IL-2 | 0.52(0.41, 0.90) | 1.07(1.00, 1.31) | 0.002 |
| IL-4 | 1.07(0.67, 1.25) | 1.54(1.36, 1.76) | 0.005 |
| TNF-α | 0.89(0.69, 1.23) | 1.43(1.23, 1.67) | 0.011 |
| IL-5 | 0.33(0.00, 0.19) | 0.31(0.28, 0.49) | 0.006 |
| IL-12 | 1.09(0.45, 1.56) | 1.80(1.49, 2.36) | 0.004 |

Median and interquartile range from cytometric bead array are displayed. All parameters are expressed in pg/mL.
